# Acyl-CoA N-acyltransferase influences fertility by regulating lipid metabolism and jasmonic acid biogenesis in cotton

**DOI:** 10.1038/srep11790

**Published:** 2015-07-02

**Authors:** Wenfeng Fu, Ying Shen, Juan Hao, Jianyong Wu, Liping Ke, Caiyun Wu, Kai Huang, Binglun Luo, Mingfeng Xu, Xiaofei Cheng, Xueping Zhou, Jie Sun, Chaozhu Xing, Yuqiang Sun

**Affiliations:** 1 College of Life Sciences, Zhejiang Sci-Tech University, Hangzhou, 310018, Zhejiang, China; 2 College of Life and Environmental Science, Hangzhou Normal University, Hangzhou, 310016, Zhejiang, China; 3 State Key Laboratory of Cotton Biology, the Institute of Cotton Research of CAAS, Anyang, 455000, Henan, China; 4 State Key Laboratory for Biology of Plant Diseases and Insect Pests, Institute of Plant Protection of CAAS, Beijing, 100193, China; 5 The Key Laboratory of Oasis Eco-agriculture, College of Agriculture, Shihezi University, Shihezi, 832000, Xinjiang Province, China

## Abstract

Cotton (*Gossypium* spp.) is an important economic crop and there is obvious heterosis in cotton, fertility has played an important role in this heterosis. However, the genes that exhibit critical roles in anther development and fertility are not well understood. Here, we report an acyl-CoA N-acyltransferase (EC2.3; GhACNAT) that plays a key role in anther development and fertility. Suppression of *GhACNAT* by virus-induced gene silencing in transgenic cotton (*G. hirsutum* L. cv. C312) resulted in indehiscent anthers that were full of pollen, diminished filaments and stamens, and plant sterility. We found GhACNAT was involved in lipid metabolism and jasmonic acid (JA) biosynthesis. The genes differentially expressed in *GhACNAT*-silenced plants and C312 were mainly involved in catalytic activity and transcription regulator activity in lipid metabolism. In *GhACNAT*-silenced plants, the expression levels of genes involved in lipid metabolism and jasmonic acid biosynthesis were significantly changed, the amount of JA in leaves and reproductive organs was significantly decreased compared with the amounts in C312. Treatments with exogenous methyl jasmonate rescued anther dehiscence and pollen release in *GhACNAT*-silenced plants and caused self-fertility. The *GhACNAT* gene may play an important role in controlling cotton fertility by regulating the pathways of lipid synthesis and JA biogenesis.

Cotton (*Gossypium hirsutum* L.) has long been the world’s most important source of natural textile fiber and is one of the first genetically modified crops. The modifications include transgene-mediated herbicide tolerance and pest-resistance^1^. Biotechnology has now accelerated cotton breeding programs, especially heterosis development. *G. hirsutum* is allotetraploid with a large and complex genome (AADD, 2n = 4x = 52); thus, it is difficult to study gene function in cotton with traditional methods of RNA interference. This approach is hindered by the inefficient production of stable transformed plants because of the long periods of plant tissue culture and the very low efficiency of transformation, which is genotype limited^1^. The genome of the wild cotton species *G. raimondii* was sequenced and showed many genes involved in cotton fiber initiation and elongation in the DD genome^2^. Another study analyzed the polyploidization of *Gossypium* genomes and the evolution of spinnable cotton fibers^3^. The genome sequence of *G. arboreum* has been completed recently, providing information about the AA genome^4^. In public databases, an increasing number of expressed sequence tags (ESTs) and deep sequencing data for cotton have become available. A rapid and cost-effective tool has been needed to connect the gene sequence information with gene function in cotton. As a transient, efficient expression tool, virus-induced gene silencing (VIGS) has been used in many species, and there are different vector systems available for the different species^5^.

Transient transformation with a specific virus has become a fast and efficient method to analyze gene function at the genome-wide level. VIGS mediated by *Agrobacterium* is an efficient, transient posttranscriptional gene silencing method for fast, low-cost and large-scale gene function analysis in plants. VIGS exploits the innate plant defense system of posttranscriptional gene silencing (PTGS) against intracellular viral proliferation and extracellular viral movement^6^. Both RNA and DNA plant viruses can contribute to VIGS^5^, including the viral vectors derived from positive-strand RNA, *Potato virus X* (PVX), *Tobacco mosaic virus* (TMV), and *Tobacco rattle virus* (TRV)^7^. Different sources of VIGS vectors are the bipartite single-strand DNA viruses and the mono-partite DNA viruses that may require a helper virus or satellite DNA for inducing disease symptoms, including the *Tomato yellow leaf curl China virus* (TYLCCV)^5^,^7^,^8^. Currently, both types of VIGS viruses, including TRV^9^,^10^ and *Cotton leaf crumple virus* (CLCrV) based silencing vectors^11^, have been used in the analysis of cotton gene function. The CLCrV is a member of the genus Begomovirus, family *Geminiviridae*, and it consists of two circular single-stranded DNAs called CLCrV DNA-A and CLCrV DNA-B with different, indispensable functions^12^,^13^,^14^. The new, modified CLCrV-based vector, pCLCrV, was established specifically for cotton infection^15^. In this study, we utilized the pCLCrV-VIGS system to analyze the function of the *GhACNAT* gene. The *GhACNAT* gene was acquired from the differentially expressed genes of *G. hirsutum* L. cv. C312 and its male sterile mutant *ms1*. The *ms1* mutant has the same phenotype and genetic background as C312 except for male sterility. Using cDNA-AFLP, we screened for genes that showed differential expression between C312 and *ms1* to obtain the genes related to male sterility. The *GhACNAT* gene was significantly differentially expressed with an unknown function in male sterility. The *GhACNAT* gene is homologous to the *Arabidopsis thaliana* (*A. thaliana*) gene *At2g23390*, which is a member of the superfamily that encodes acyl-CoA N-acyltransferase (EC2.3).

N-Acyltransferase is one of the important enzymes that catalyze the transfer of an acyl group to a substrate; N-acyltransferase is implicated in a variety of functions and it is conserved from bacterial antibiotic resistance to circadian rhythms in mammals. Members of the N-acyltransferase superfamily have a similar catalytic mechanism but vary in the types of acyl groups they transfer, including those of the three main nutrient substances, saccharides, lipids and proteins. These substances participate in a common metabolic pathway mediated by acetyl-CoA in the tricarboxylic acid cycle and oxidative phosphorylation reactions, which involve a variety of enzymes.

Acyl lipids have various functions in plants, and the structures and properties of the acyl lipids vary greatly even though they are all derived from the same fatty acid and glycerolipid biosynthesis pathway^16^. Some of them, including jasmonic acid, participate in signaling pathways^17^.

Acyl-CoA and acyl-CoA N-acyltransferase are involved in these metabolic pathways, including pyruvate dehydrogenase and pyruvate formate lyase reactions, and they are involved in the metabolism of sugars in the citric acid cycle and fatty acids and fat metabolism required for the synthesis of flavonoids and related polyketides for the elongation of fatty acids involved in sesquiterpenes, brassinosteroids, and membrane sterols.

The *AtGPAT1* (glycerol-3-phosphate acyltransferase) gene has been identified as a member of the membrane-bound glycerol-3-phosphate acyltransferase gene family, correlated with several fatty acids produced in flower tissues and seeds, including linoleic acid (18:2). Jasmonic acid is derived from linolenic acid (LA)^18^, and its biosynthesis is catalyzed by several enzymes, including lipoxygenase (LOX), allene oxide synthase (AOS), allene oxide cyclase (AOC), and 12-oxo-phytodienoic acid reductase (OPR) followed by three cycles of β-oxidation^19^,^20^,^21^. The mutants of *opr3*, *dad1* (*defective in anther dehiscence1*), *ms35* (*male sterile35*) and the genes *AOS*, *AOC*, *AIF* (ANTHER INDEHISCENCE FACTOR) in *A. thaliana* and other species (tomato, tobacco, brassica, rice and maize) regulate the male or female sterility involved in JA biosynthesis^22^,^23^,^24^,^25^,^26^,^27^,^28^. The *AOC* gene is highly expressed in distinct flower organs and vascular bundles and in jasmonic acid influenced female fertility in the tomato^27^. The *OPR7* and *OPR8* genes regulate the diverse functions of JA in maize^29^. In *G. hirsutum*, *GhSERK1* (somatic embryogenesis receptor-like kinase) has been shown to regulate pollen development^30^.

Lipid metabolism plays a very important role in the development of the tapetum and pollen grains^31^. We constructed the pCLCrV*-GhACNAT* vector to silence the target gene *GhACNAT* in *G. hirsutum* cv. C312. In the transgenic *GhACNAT*-silenced plants, the phenotype of the reproductive organs was investigated. Genes that were expressed at different levels in C312 and *GhACNAT*-silenced plants were screened and assayed by qRT-PCR to study the function of *GhACNAT* and related genes. The results indicated that *GhACNAT* played an important role in regulating the development of reproductive organs and fertility by influencing lipid and JA biosynthesis.

## Results

### *GhACNAT* is differentially expressed in the male sterile *ms*1 mutant and C312

**Figure 1:**
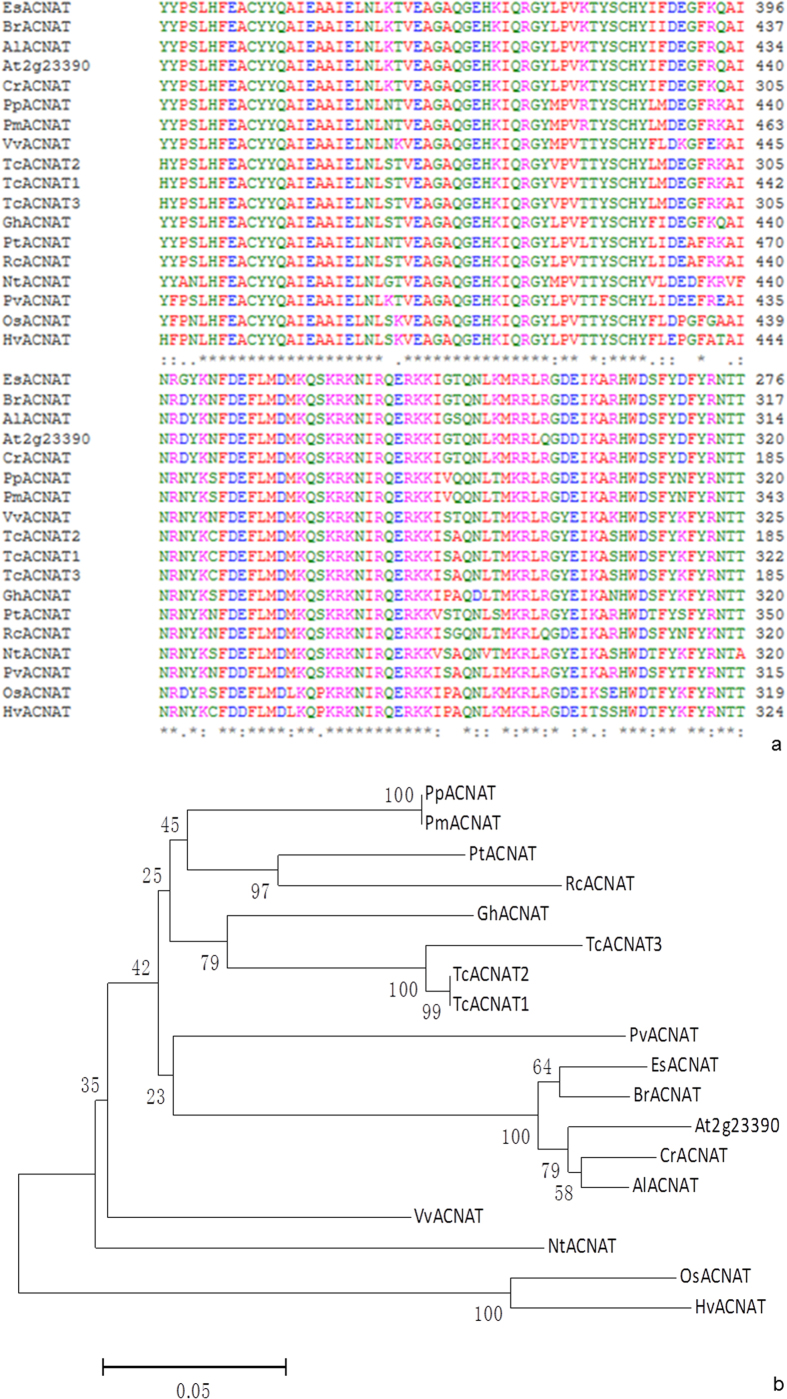
Phylogenetic analysis of the ACNAT family. (**a**) GhACNAT had high homology with ACNAT in different species. (**b**) The phylogenetic cladogram of the ACNAT superfamily in different species. Pp, *Prunus persica* (XP_007222936.1); Tc1, *Theobroma cacao*1 (XP_007022847.1); Tc2, *Theobroma cacao*2 (XP_007022848.1); Tc3, *Theobroma cacao*3 (XP_007022849.1); Pm, *Prunus mume* (XP_008218653.1); Vv, *Vitis vinifera* (XP_002268159.2); Pt, *Populus trichocarpa* (XP_006385079.1); Es, *Eutrema salsugineum* (XP_006402222.1); Cr, *Capsella rubella* (XP_006294555.1); Br, *Brassica rapa* (XP_009134028.1); Nt, *Nicotiana tomentosiformis* (XP_009618447.1); Pv, *Phaseolus vulgaris* (XP_007153914.1); Os, *Oryza sativa* Japonica Group (NP_001052881.1); Hv, *Hordeum vulgare subsp. vulgare* (BAJ94372.1); Rc, *Ricinus communis* (XP_002527856.1); Al, *Arabidopsis lyrata subsp. Lyrata* (XP_002880496.1); At, *Arabidopsis thaliana* (At2g23390.1); Gh, *G. hirsutum* L.

Among the genes differentially expressed in *G. hirsutum* cv. C312 and the male sterile *ms1* mutant of C312, the acyl-CoA N-acyltransferase (EC2.3) gene was significantly down-regulated with unknown function and designated *GhACNAT*. *GhACNAT* is homologous to genes in different species, including with *At2g23390* (Fig. 1a), which is a member of the acyl-CoA N-acyltransferase superfamily in *A. thaliana* with a conserved domain that catalyzes N-acetyltransferase reactions in lipid synthesis and metabolism (Fig. 1b). We amplified the full-length 1410 bp cDNA of *GhACNAT* from C312. One highly homologous gene was found in the DD genome of *G. raimondii*.

### The expression pattern of the *GhACNAT* gene analyzed by qRT-PCR

**Figure 2:**
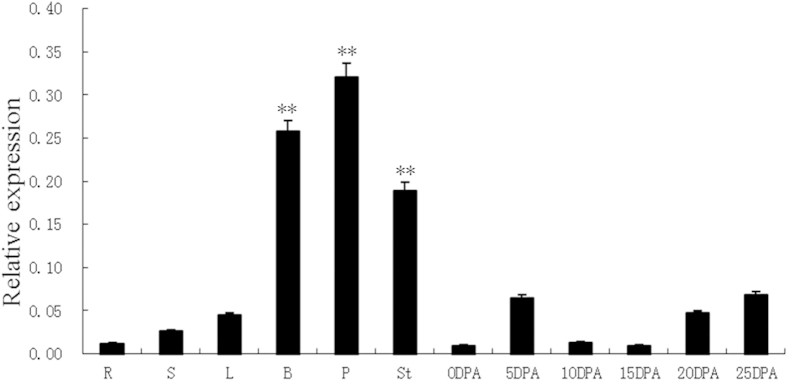
The GhACNAT gene expression pattern in the cotton variety C312. R: root; S: stem; L: leaves, B: bract, P: petals, St: stigmas and stamens, and fibers of different development stages (0-, 5-, 10-, 15-, 20, 25-DPA) in C312. (DPA: days post anthesis). The values are the means±s.ds. for three biological replicates. The asterisks indicate statistically significant differences between the transgenic and WT plants (*P < 0.05, **P < 0.01, Student’s t-test).

The *GhACNAT* gene was expressed at different levels in the tested tissues and organs, including roots, stems, leaves, bracts, petals, stamens stigmas, and fibers of different development stages (0-, 5-, 10-, 15-, 20-, and 25-DPA (days post anthesis)) in C312 (Fig. 2). The expression levels of the *GhACNAT* gene in the reproductive organs (bracts, petals, stigmas and stamens) were significantly higher than in the vegetative organs (roots, stems, and leaves) and fibers of different developmental stages. The *GhACNAT* gene maintained significantly high-level expression in the flower organs, including the sepals, petals, stamens and stigmas.

### The construction of the pCLCrV-*GhACNAT* vector for VIGS

The pCLCrV-based virus-induced gene-silencing system produced an efficient and persistent gene silencing in cotton. To determine whether the pCLCrV-based silencing vector could silence endogenous gene expression in cotton, a 327 bp fragment of the phytoene desaturase gene (*PDS*), commonly used as a positive marker for VIGS, was cloned into the pCLCrVA vector to construct pCLCrV-*PDS*. *Agrobacteria tumefaciens* strain GV3101 containing different pCLCrVA and pCLCrVB vector was infiltrated by syringe into the cotyledons of 2-week-old seedlings of C312. By 20 days postinfiltration, cotton plants infiltrated with *Agrobacteria* harboring both pCLCrVA-*PDS* and pCLCrVB showed the typical *PDS* gene-silencing phenotype of photobleaching in newly emerging leaves.

The photobleaching phenotype in plants infiltrated with pCLCrV-*PDS* was observed on the newly emerging leaves by 3 weeks postinfiltration, first appearing in the nearby veins and gradually extending to the whole leaves, which became mosaic with green spots (Fig. S1). The efficiency of the photobleaching in the plants infiltrated with pCLCrV-*PDS* and pCLCrVB was greater than 75%. The transgenic pCLCrV-*PDS* plants were confirmed by PCR and showed the photobleaching mosaic with green spots in the leaves. Transgenic plants of the pCLCrVA-empty with pCLCrVB and pCLCrVA-*GhACNAT* with pCLCrVB showed a slight shrinking of the new leaves (Fig. S1 a, b, c). The transgenic plants with pCLCrVA-empty and pCLCrVA-*GhACNAT* were identified by PCR with specific primers (Table S3). The photobleaching in the transgenic pCLCrV-*PDS* plants appeared in different organs, including the stem, petiole, bracts, and bolls during the growth cycle (Fig. S1), indicating that the silencing caused by this vector persisted in the cotton plants. Pink stripes appeared on the stems in the transgenic pCLCrV-*PDS* plants (Fig. S1 e). To investigate the silencing efficiency, the levels of *PDS* mRNA in both pCLCrV-*PDS*-silenced and wild-type C312 plants were measured by quantitative real time PCR (qRT-PCR). In the transgenic pCLCrV-*PDS* plants with strong photobleaching of the new leaves, the expression level of the endogenous *PDS* gene was significantly reduced, 2–40-fold compared with wild-type C312 plants (Fig S1i). The control plants of C312 and pCLCrV-empty did not show the photobleaching phenotype at any time during the studies (Fig S1c).

A 200 bp fragment of the *GhACNAT* gene was amplified to construct the pCLCrV-*GhACNAT* vector to silence the endogenous *GhACNAT* gene in C312 plants.

### Silencing the *GhACNAT* gene influenced the development of the reproductive organs of transgenic plants

**Figure 3:**
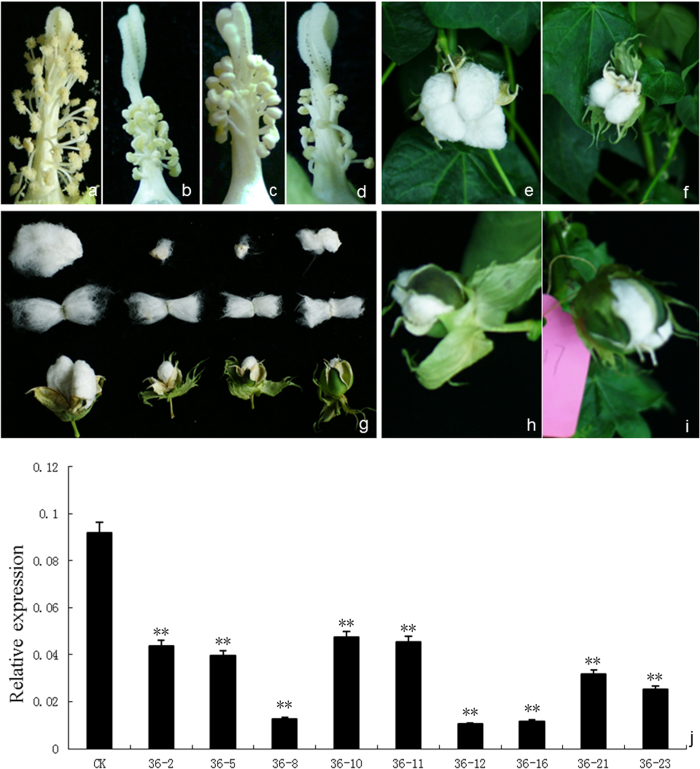
The morphological changes and *GhACNAT* expression analysis in transgenic *GhACNAT*-silenced plants and C312 plants. (**a-d**) the stamens and stigmas of C312 (**a**) and transgenic *GhACNAT*-silenced plants (**b, c, d**); (**e-i**) bolls of C312 (**e**) and transgenic *GhACNAT*-silenced plants pollinated with C312 pollen (**f, h, i**); (**g**) fibers and seeds of C312 and transgenic *GhACNAT*-silenced plants from left to right. (**j**) Relative expression levels of *GhACNAT* measured by qRT-PCR in leaves of *GhACNAT*-silenced plants (lines 36-2, 36-5, 36-8, 36-10, 36-11, 36-12, 36-16, 36-21 and 36-23) and in C312 plants. The *Ubiquitin7* gene (*GhUBQ7*) was used as an internal control. The values are the means for three replicates (samples of each line collected on days 20, 35 and 50 post-infection). The asterisks indicate statistically significant differences between the transgenic *GhACNAT*-silenced and WT C312 plants (Significant *P < 0.05, highly significant **P < 0.01, Student’s t-test).

**Figure 4:**
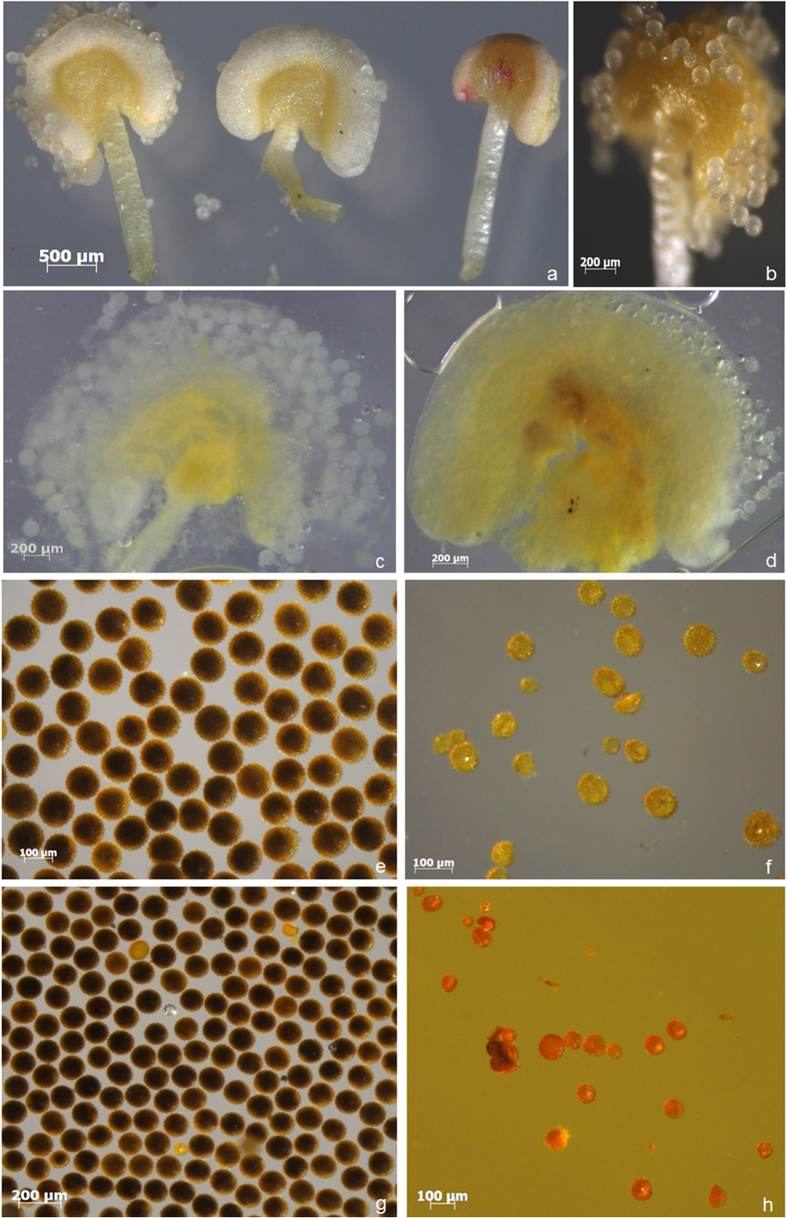
Phenotypic analysis of anther and pollen in WT C312 plants, transgenic *GhACNAT*-silenced plants and male sterile *ms*1 plants. (**a**) comparison of anther and pollen in C312, transgenic *GhACNAT*-silenced plants and *ms1* plants (from left to right); (**b**) pollen releasing from the anther in C312; (**c**) anther of C312 plant dehiscent and full of pollen grains; (**d**) indehiscent anther of the sterile transgenic *GhACNAT*-silenced plant, indehiscent and full of pollen grains; (**e**) 99% of the active pollen of WT C312 was stained with KI-I_2_; (f, h) the pollen of *ms1* plants with no activity stained with KI-I_2_; (**g**) 95% of the active pollen of the transgenic *GhACNAT*-silenced plants were stained with KI-I_2_.

There were no obvious differences in the phenotypes of the control plants, the transgenic pCLCrV-*PDS* plants and the transgenic *GhACNAT*-silenced plants during vegetative growth except for the mosaic photobleaching spots in the transgenic pCLCrV-*PDS* plants. In the reproductive stage of the transgenic *GhACNAT*-silenced plants, the distinct phenotypic changes appeared in the reproductive organs (Fig. 3). The stigmas were forked and warped compared with the stigmas of C312 (Fig. 3a) and the transgenic control plants. The stamens of the transgenic *GhACNAT*-silenced plants appeared indehiscent but full of viable pollen (Fig. 3b,c,d and 4a,d,g). The filaments became shorter, and the number of stamens decreased significantly (Table S1). No pollen was released from the closed anthers in the transgenic *GhACNAT*-silenced plants. In the male sterile *ms1* mutant, the anthers were closed and shrunken, and no pollen formed (Fig. 4a,f,h). The anthers of the C312 plants were dehiscent and full of active pollen grains, whereas the stamens of the transgenic *GhACNAT*-silenced plants were indehiscent but as full of viable pollen as the wild-type C312 (Fig. 4d,g). However, the pollen could not be released from the closed stamens. In the transgenic *GhACNAT*-silenced plants, mature bolls were obtained successfully by pollination with normal C312 pollen. The bolls had two to four loculi containing seeds covered with fibers (Fig. 3e–i).

In the transgenic pCLCrV-*GhACNAT*-silenced plants, the endogenous *GhACNAT* gene was significantly down-regulated (2–12-fold) compared with C312 (Fig. 3j). The lines designated 36-8, 36-12 and 36-16 were selected for further analysis of the phenotypes and expression changes of related genes.

### The analysis of genes differentially expressed in transgenic *GhACNAT*-silenced plants and control plants

**Figure 5:**
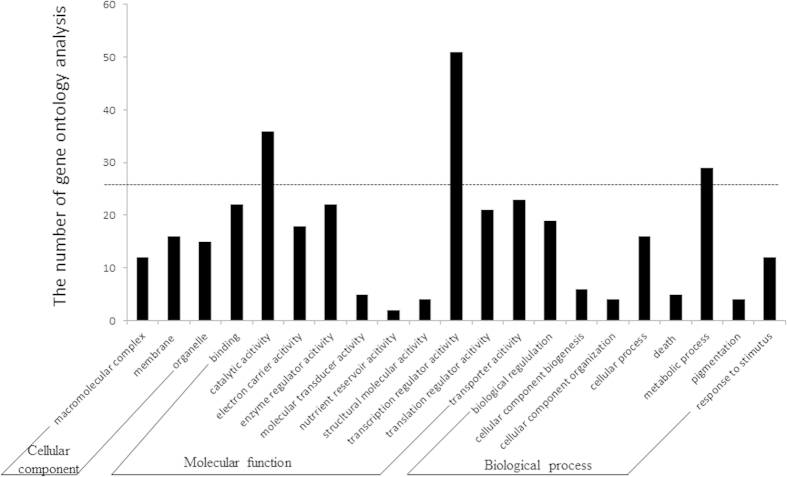
Functional classification into different categories of differentially expressed fragments from reproductive organs of pCLCrV-empty and transgenic *GhACNAT*-silenced plants.

To study differentially expressed genes related to the alteration of reproductive organs in the transgenic *GhACNAT*-silenced plants, 3 individual plants of wild type C312 and the transgenic pCLCrV*-GhACNAT*-silenced lines were selected for differential expression analysis by cDNA-AFLP. The stigmas and stamens of the flowers (on the day of anthesis) were picked and collected for total RNA isolation. The differentially expressed genes were selected, and 450 fragments (100–200 bp) were cloned and sequenced. The function annotations of the differentially expressed genes in the reproductive organs of the control plants and the transgenic *GhACNAT*-silenced plants were divided into several categories (Fig. 5). The main differentially expressed genes were focused on catalytic activity, translation regulator activity and metabolic processes involved in lipid metabolism. Acyl-CoA and acyl-CoA N-acyltransferase were involved in the lipid metabolic pathways.

### The expression changes of genes related to the development of reproductive organs in the transgenic *GhACNAT*-silenced plants

Many mutants with anther indehiscence, including those with mutations in *opr3*^22^ and *dad1*^23^, showed that jasmonic acid participates in the regulation of anther development, filament elongation and pollen viability^32^. *AtAIF* was identified as a repressor that controls anther dehiscence by regulating the jasmonic acid biosynthesis pathway in *A. thaliana*^24^. Linolenic acid (18:3) was released from membrane phospholipid by a lipolytic enzyme (DAD1), and the *DAD1* gene participated in the regulation of anther development^23^.

**Figure 6:**
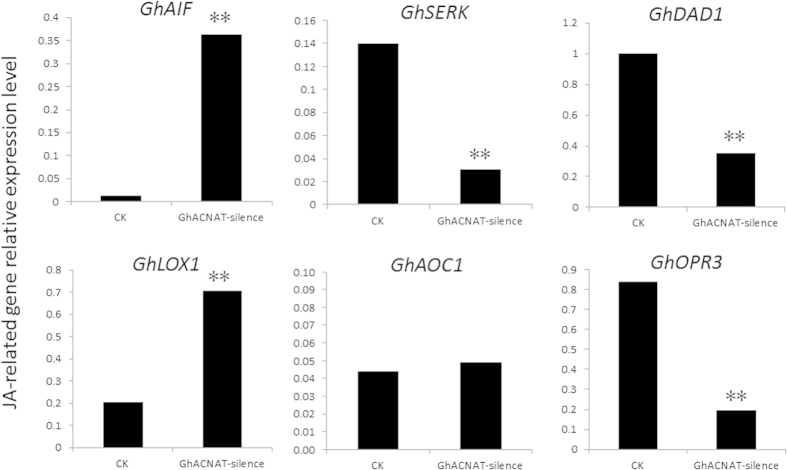
The relative expression of genes related to anther development and JA biosynthesis by qRT-PCR in transgenic *GhACNAT*-silenced plants and in C312 as a control (CK). The *Ubiquitin7* gene (*GhUBQ7*) was used as an internal control. The values are the means for three replicates (one flower for each sample of each line). The asterisks indicate statistically significant differences between the transgenic *GhACNAT*-silenced and the WT plants (*P < 0.05, **P < 0.01, Student’s t-test).

JA plays a role in anther development. The expression levels of genes encoding key enzymes involved in the JA biosynthesis pathway were examined in transgenic *GhACNAT*-silenced plants and C312 plants. The genes (*GhAIF*, *GhDAD1*, *GhLOX1* and *GhOPR3*) with high homology to *AtAIF*, *AtDAD1, AtLOX1, and AtOPR3* in *A. thaliana*, which are genes related to reproductive organ development, were detected by qRT-PCR in stigmas and stamens of the C312 plants and the transgenic *GhACNAT*-silenced plants on the blossom day (Fig. 6). *GhAIF* was significantly up-regulated (29-fold) in the transgenic *GhACNAT*-silenced plants compared with the control plants. The *GhLOX1* gene was up-regulated 3.5-fold compared with the gene in control plants. The corresponding enzyme catalyzes the conversion of linolenic acid to 13-hydroperoxylinoinic acid, which is the precursor for JA, and *cis*-3 hexenal+traumatin using two different and competing pathways. The expression of *GhAOC1* was not significantly different in C312 and transgenic *GhACNAT*-silenced plants. The expression of the *GhDAD1* gene was significantly reduced in *GhACNAT*-silenced plants, and the expression of the *GhOPR3* gene for JA biosynthesis was reduced approximately 4.5-fold in transgenic *GhACNAT*-silenced plants. This showed that JA biogenesis was influenced by changes in expression of these genes, which could cause anther indehiscence and short filaments. The *GhSERK* gene, which was shown to regulate anther dehiscence and pollen viability in cotton^30^, was identified from a differentially expressed fragment by cDNA-AFLP. The expression of *GhSERK* was significantly reduced in the stamens of the transgenic *GhACNAT*-silenced plants.

### The *GhACNAT* silencing influenced fatty acid biosynthesis in the transgenic plants

**Figure 7:**
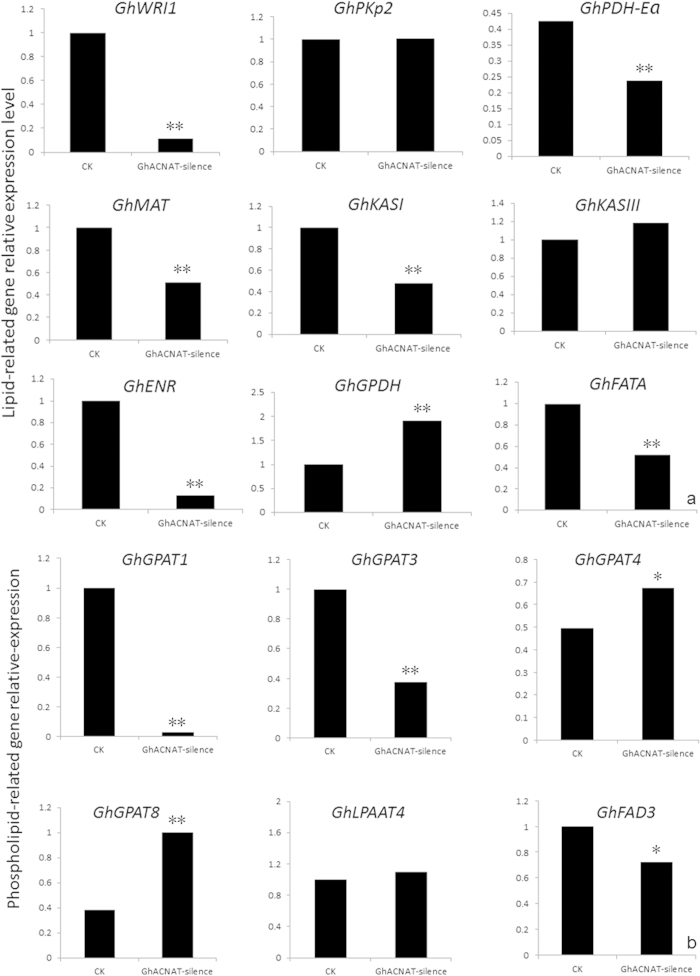
The relative expression of genes participating in the lipid biosynthesis pathway. (**a**) The relative expression of the main genes for fatty acid synthesis; (**b**) the relative expression of genes for glycerolipid biosynthesis. The values are the means for three replicates (one flower for each sample of each line). The asterisks indicate statistically significant differences between the transgenic *GhACNAT*-silenced and the WT plants (*P < 0.05, **P < 0.01, Student’s t-test).

*GhACNAT* belongs to the gene superfamily encoding acyl-CoA N-tranferase and has high homology with the *At2g23390* gene. The acyl-CoA N-tranferase superfamily primarily participates in lipid metabolism to provide acyl-CoA. To investigate the function of *GhACNAT* in fatty acid biosynthesis, several key genes in the fatty acid biosynthetic pathway were measured by qRT-PCR. The single transcription factor WRI1, which has been shown to directly control the transcriptional activation of the fatty acid biosynthetic pathway^33^, was significantly decreased in the transgenic *GhACNAT*-silenced plants. The glycolytic and late fatty acid biosynthetic genes, including *GhPDH-E1a* (homologous to *At1g01090*), *GhBCCP2* (homologous to *At5g15530*), *GhMAT* (homologous to *At2g30200*), *GhKASI* (homologous to *At5g46290*), *GhENR* (homologous to *At2g05990*), and *GhFATA* (homologous to *At3g25110*), were measured in the stigmas and stamens of the control plants and the transgenic *GhACNAT*-silenced plants (Fig. 7a). In the transgenic *GhACNAT*-silenced plants, the expression levels of *GhPDH-E1a, GhBCCP2*, and *GhMAT* were reduced 2-fold compared with the control plants, and the expression levels of *GhKASI* and *GhENR* were significantly down-regulated. The relative mRNA levels of *GhCYP86A2* (homologous to *At4g00360*), *GhCYP86A8* (homologous to *At2g45970*), and *GhCYP77A6* (homologous to *At3g10570*), which are involved in cuticle formation, were almost the same in the transgenic *GhACNAT*-silenced plants and the control plants. In the *GhACNAT*-silenced plants, the expression of the genes involved in lipid synthesis was reduced significantly, and the tendency was consistent. The changes in gene expression influenced lipid synthesis and reduced JA biosynthesis in the stigmas and stamens of the *GhACNAT*-silenced plants.

The *AtGPAT1* gene is associated with several fatty acid composition changes in flower tissues and seeds, including JA biosynthesis, catalyzed by lipoxygenase (LOX), allene oxide synthase (AOS), allene oxide cyclase (AOC), and 12-oxo-phytodienoic acid reductase (OPR). The genes *GhGPAT1*, *GhGPAT3* and *GhGPAT*4 were detected by qRT-PCR and showed obvious differences in expression in C312 and the transgenic *GhACNAT*-silenced plants. The expression levels of *GhGPAT1* and *GhGPAT3* were significantly decreased, whereas *GhGPAT4* expression increased in the transgenic *GhACNAT*-silenced plants. *GhGPAT*, *GhLPAAT* and *GhFAD3* participate in the synthesis of phospholipids. In the transgenic *GhACNAT*-silenced plants, the expression of *GhLPAAT* did not change, but *GhFAD3* expression decreased (Fig. 7b). In the JA biosynthesis pathway from the acyl-CoA pool to phospholipid to linolenic acid, the expression of genes encoding key enzymes significantly changed to influence JA biosynthesis.

### Measurement of JA and rescue of transgenic *GhACNAT*-silenced plants treated with exogenous MeJA

**Figure 8:**
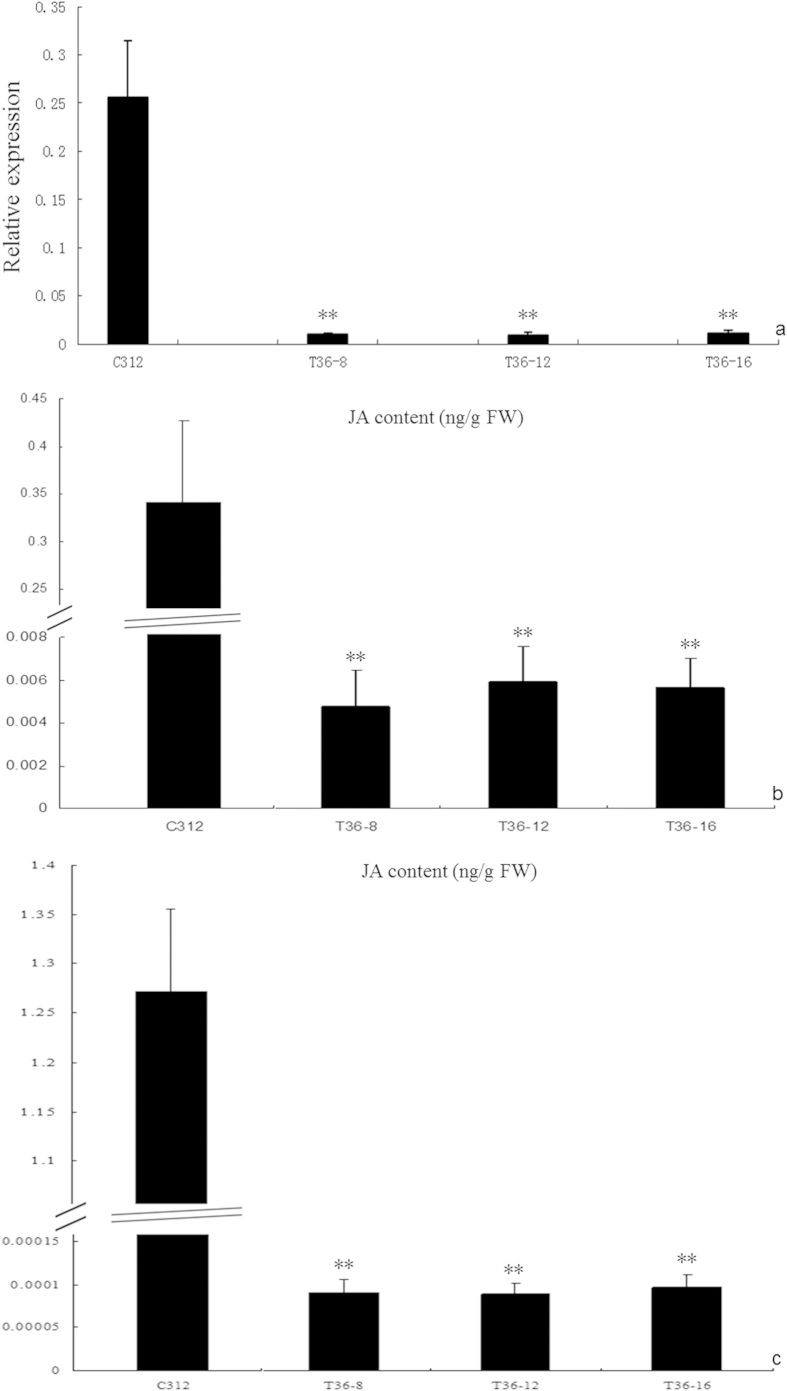
The relative expression of the *GhACNAT* gene in reproductive organs (**a**), and JA levels in C312 and transgenic *GhACNAT*-silenced plants in leaves (**b**) and in the whole flowers (**c**). The values are the means±s.ds for three replicates (three leaves for b and three flowers of each line for a and c). The asterisks indicate statistically significant differences between the transgenic *GhACNAT*-silenced and the WT plants (*P < 0.05, **P < 0.01, Student’s t-test).

Endogenous *GhACNAT* expression was significantly decreased by VIGS in the reproductive organs of *GhACNAT*-silenced plants (Fig. 8a), and it significantly influenced the expression of genes related to JA biosynthesis (Fig. 6) and caused abnormal formation of reproductive organs. In transgenic *GhACNA*T-silenced plants, the genes for repressing JA biosynthesis were up-regulated, whereas the expression levels of genes involved in JA biosynthesis were down-regulated and decreased the JA levels in leaves and reproductive organs (Fig. 8b,c). The amounts of JA in fresh leaves of three transgenic *GhACNAT*-silenced lines were significantly lower than in C312, and in the reproductive organs, the amounts of JA were all higher than those in leaves. In the flowers of three *GhACNAT*-silenced lines, the amounts of JA were extremely low or even undetectable by GC/MS (Fig S3) and significantly lower than in C312. However, the transcripts of genes for JA biosynthesis were apparently attenuated in transgenic *GhACNAT*-silenced lines compared with C312 plants, and the genes for repressing JA biosynthesis were up-regulated (Fig. 6). Consistent with the kinetics of gene expression in the JA pathway, JA levels in the leaves and reproductive organs in transgenic *GhACNAT*-silenced plants were dramatically decreased compared with C312 plants (Fig. 8b,c).

**Figure 9:**
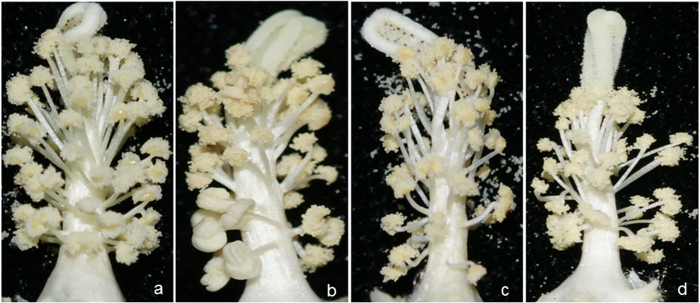
The morphologies of stamens and stigmas in C312 and transgenic *GhACNAT*-silenced plants treated with different concentrations of MeJA. (**a**) normal stamens and stigma in C312 plants; (**b**) stamens and stigma in transgenic *GhACNAT*-silenced plants treated with 100 μM MeJA; (**c**) stamens and stigma in transgenic *GhACNAT*-silenced plants treated with 500 μM MeJA; (**d**) stamens and stigma in transgenic *GhACNAT*-silenced plants treated with 1000 μM MeJA.

For the rescue experiment in transgenic *GhACNAT*-silenced plants to determine whether the defect in jasmonic acid synthesis caused the anther dehiscence, three concentrations (100 μM, 500 μM and 1000 μM) of MeJA were used to treat the buds of transgenic *GhACNAT*-silenced plants before they opened. In the opening flowers of transgenic *GhACNAT*-silenced plants treated with MeJA, the morphologic changes in the reproductive organs were reversed, and the anthers were thoroughly or partially dehiscent, similar to the normal anthers in C312 plants (Fig. 9, Table S2). With 100 μM MeJA, the anthers partially dehisced, and some anthers were still occlusive (Fig. 9b; Table S2); with 500 μM and 1000 μM MeJA, all the anthers dehisced normally (Fig. 9c,d; Table S2). The bolls of transgenic *GhACNAT*-silenced plants were obtained by self-pollination after treatment with MeJA. The results showed that exogenous MeJA could reverse the anther phenotype and rescue the occlusive anthers caused by decreasing endogenous JA.

## Discussion

### VIGS provided an efficient method to analyze gene function in cotton during the entire growth cycle

VIGS has demonstrated to be an efficient method to analyze gene function in cotton^10^,^11^,^15^,^34^. By 3 weeks after the pCLCrV-*Agrobacteria* injection, transgenic pCLCrV-*PDS* plants showed obvious photobleaching in the new leaves. The efficiency of transformation reached 75%, and the effects were retained in leaves, stems, flowers, and even in bolls throughout the growth cycle. In the vegetative growth stage, there was no obvious phenotype except for slight wrinkling in the leaves of the transgenic *GhACNAT*-silenced plants. At the stage of reproductive growth, the phenotypes of the reproductive organs changed significantly, showing stigma wraped, anther indehiscence and no pollen release, which led to sterility in the transgenic *GhACNAT*-silenced plants. The sterility was mainly caused by the anther dehiscence and failure to release pollen. The changes of phenotypes in transgenic *GhACNAT*-silenced plants demonstrated that the effect of VIGS in cotton can be used for gene function analysis in vegetative growth, in reproductive growth and even in developing fibers.

### The anther development was influenced by JA biosynthesis regulated by the *GhACNAT* gene

The *GhACNAT* gene belongs to the acyl-CoA N-acyltransferase gene superfamily and was selected from the genes differentially expressed in C312 and its male sterile mutant, *ms1*. The *GhACNAT* gene is homologous with *At2g23390* in *A. thaliana*, which encodes acyl-CoA N-acyltransferase, an enzyme involved in the isopentenyl diphosphate biosynthetic pathway, maltose metabolism, regulation of proton transport, and starch biosynthesis and was found to be expressed in vegetative organs and in reproductive organs^35^,^36^,^37^,^38^.

**Figure 10:**
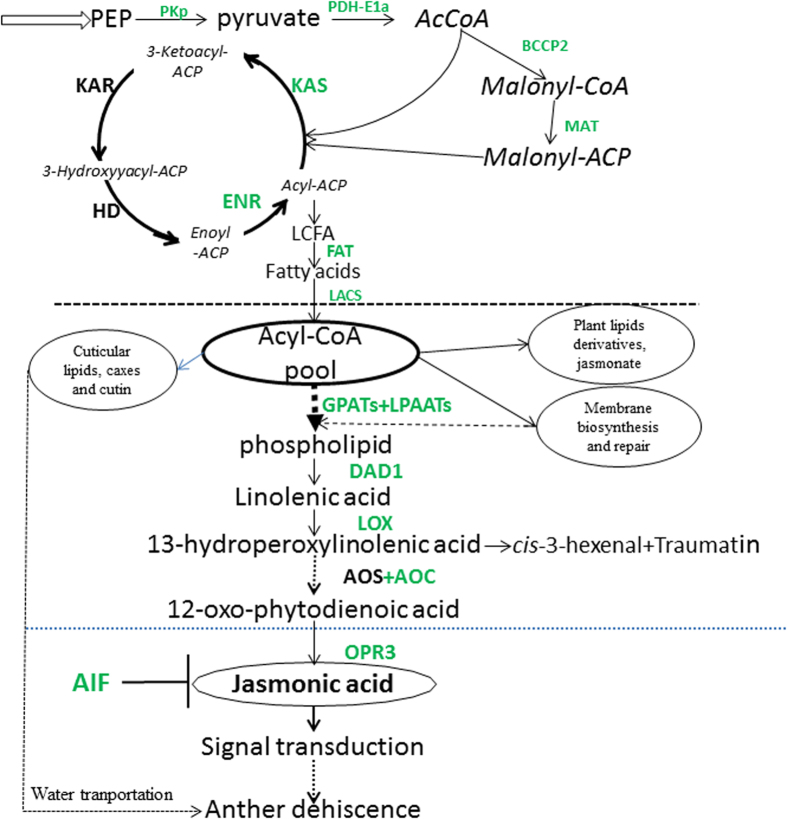
A simple schematic diagram of fatty acid and jasmonic acid biosynthesis. PEP, phosphoenolpyruvate; PKp, plastidial pyruvate kinase; PDH-E1a, pyruvate dehydrogenase; AcCoA, acetyl-CoA; ACP, acyl carrier protein; BCCP2, biotin carboxyl carrier protein2; MAT, malonyl-CoA: ACP transacylase; KAS, 3-ketoacyl-ACP synthase; KAR, 3-ketoacyl-ACP reducase; HD, 3-hydroxyacyl-ACP dehydratase; ENR, enoyl-ACP reductase; LCFA, long-chain fatty acid; LACS, long-chain acyl-CoA synthetase; FAT, fatty acyl-ACP thioesterase; GPAT, glycerol-3-phosphate acyltransferase; LPAAT, lysophosphaditic acid acyltransferase; LOX, lipoxygenase; AOS, allene oxide synthase; AOC, allene oxide cyclase.

Acyl-lipid metabolism in *A. thaliana* is a complex process and is organized into 12 sections based on the types of lipids produced and their subcellular localization^39^. Acyl lipids in plants have many diverse functions, involving more than 10 membrane lipid classes in the endoplasmic reticulum (ER), including the phospholipids, glycolipids, and sphingolipids^39^,^40^,^41^,^42^,^43^. The first committed step in fatty acid synthesis is the formation of malonyl-CoA from acetyl-CoA and bicarbonate by acetyl-CoA carboxylase (ACC)^16^,^44^. Before entering the fatty acid synthesis pathway, the malonyl-CoA group is transferred from CoA to ACP catalyzed by malonyl-CoA: acyl carrier protein malonyltransferase (MCMT). Malonyl-ACP provides two-carbon units at each step of elongation. The long-chain acyl groups hydrolyzed by acyl-ACP thioesterases release fatty acids. These fatty acids are ultimately activated to CoA esters by a long-chain acyl-CoA synthetase (LACS) and exported to the endoplasmic reticulum or possibly the PC at the plastid envelope by the action of lysophosphatidylcholine acyltransferase (LPCAT)^45^,^46^,^47^. Fatty acids are derived from plastid lipids desaturated in the ER and are precursors for potent signaling molecules, including jasmonates, leaf aldehydes or divinyl ethers^48^,^49^. The ER is the major site for phospholipid biosynthesis. Phosphatidic acid (PA) is the common precursor of phospholipids catalyzed by acyl-CoA: glycerol-3-phosphate acyltransferase (GPAT) and acyl-CoA: lysophosphatidic acid acyltransferase (LPAAT). PAs originating from the ER pathway exclusively contain C18 fatty acids in the *sn*-2 position in eukaryotic species, whereas PAs synthesized in plastids exclusively contain C16 fatty acids in the *sn*-2 position in prokaryotic species. The GPAT and LPAAT reactions of phospholipid synthesis or triacylglycerol synthesis utilize a mixed pool of acyl-CoA substrates that in many tissues is produced mostly from a PC acyl editing cycle. An *AtGPAT1* deficiency had pleiotropic effects on glycerolipid biosynthesis and resulted in several fatty acid composition changes in flower tissues and seeds. The molar proportions of palmitic acid (16:0), stearic acid (18:0), linolenic acid (18:3), and the very-long-chain eicosenoic acid (20:1) decreased to varying degrees, whereas the proportions of oleic acid (18:1) and linoleic acid (18:2) increased^31^. Linolenic acid (18:3)-derived oxylipins are the biosynthetic C18 precursors of JA^17^. Acyl groups esterified to PC are the sites of extraplastidic FA desaturation^50^. The FAD2^51^ and FAD3^52^ enzymes convert PC-bound oleate to linoleate and then linolenate, respectively, which is the precursor of JA (Fig. 10).

The *DAD1* gene encodes a lipase that hydrolyzes both TAGs (triacylglycerols) and glycerolipids^23^ and releases linolenic acid for the biosynthesis of jasmonic acid. The precursor of JA originates from an 18-carbon fatty acid of linolenic acid (LA) released by the phospholipid in membranes, which is regulated by DAD1. The biosynthesis of JA is catalyzed by the key enzymes LOX, AOS, AOC and OPR3 in *A. thaliana*. The key genes of TAG synthesis were significantly down-regulated in the stigmas and stamens of transgenic *GhACNAT*-silenced plants. The gene expression of *GhLOX* and *GhOPR3* displayed down-regulation in transgenic *GhACNAT*-silenced plants compared with the control plants. The *GhAIF* gene was identified as an inhibiting factor for JA biosynthesis^24^, and the expression level of *GhAIF* was significantly up-regulated in transgenic *GhACNAT*-silence plants.

The JA pathway was influenced because *GhACNAT*-silencing changed the expression levels of some key genes related to lipid and JA biological processes. The change in JA biosynthesis in transgenic *GhACNAT*-silenced plants resulted in the amount of JA decreasing, and the reduced JA caused anther indehiscence and other reproductive organ abnormalities. The expression changes of *GhACNAT* silenced by VIGS influenced many genes involved in biological processes of lipid and jasmonate acid. The *GhACNAT* gene, and related genes involved in biological processes of lipid and jasmonate acid, regulated the development of reproductive organs and fertility by regulating lipid metabolism and JA biosynthesis.

Future work should be focused on the application of stable knockdown lines of *GhACNAT*, including RNA interference with indihescent anthers full of active pollen, such as the transgenic *GhACNAT*-silenced plants here. The stable *GhACNAT*-silenced plant could be used to develop two-line hybrid cotton breeding using an anther indehiscent full of pollen as a sterile line and an anther releasing pollen through MeJA treatment as a maintainer line.

## Material and Methods

### Plant materials

The *G. hirsutum* L. cv. Coker 312 (C312) was used in these experiments. Seeds were germinated and grown in a greenhouse at 28 °C with a 14 h light and 10 h dark cycle. Seedlings with a 2^nd^ true leaf emergence were used for agroinfiltration. Infiltrated plants were grown in a growth chamber at 23–25 °C with a 14/10 h light/dark photoperiod.

### Gene cloning and construction of VIGS vectors

The candidate gene was obtained from the differentially expressed genes in C312 and its male sterility mutant (*ms1*). The mutant *ms1* had the same background as C312 except for the mutation. The full-length 1410 bp cDNA of the *GhACNAT* gene was amplified from the cDNA library by PCR using the forward primer 5’-CTATGGCGGCAGCACTCA-3’ and the reverse primer 5’-TGCACGATGGCTTTTCAA-3’. The PCR was incubated at 95 °C for 5 min and for 32 cycles at 95 °C for 30 s, 55 °C for 30 s, 72 °C for 100 s, and 72 °C for 10 min. The reaction was then kept at 4 °C. The PCR product was cloned into the pGEM-T Easy Vector (Promega, Madison, WI, USA), and the product was transformed into *E.coli* DH5α and sequenced.

The new CLCrV-based vector was modified from the CLCrV DNA-A and DNA-B components individually, which were inserted into the pCambia1300 vector to generate pCLCrVA and pCLCrVB, respectively^15^. The pCLCrVB is a geminivirus member and essential for the silence system in cooperation with pCLCrVA. The fragments of candidate genes were inserted into pCLCrVA to produce pCLCrVA-*GhACNAT* for VIGS in cotton plants as described by Gu *et al*., (2014)^15^.

A 200 bp fragment of the candidate gene *GhACNAT* was amplified by PCR (95 °C for 5 min and 30 cycles at 95 °C for 30 s, 59 °C for 30 s, 72 °C for 40 s, and 72 °C for 10 min. The reaction was then kept at 4 °C). The *phytoene desaturase* gene (*PDS*) causes loss of chlorophyll and carotenoids^53^,^54^ and was used as a positive marker to visualize the timing and extent of endogenous gene silencing. A 327 bp fragment of the *PDS* gene isolated from C312 by PCR (95 °C for 5 min, 30 cycles at 95 °C for 30 s, 59 °C for 30 s, 72 °C for 40 s, and 72 °C for 10 min, then 4 °C). The fragments were inserted into the pGEM-T Easy Vector (Promega, Madison, WI, USA), and the construct was transformed into *E.coli* DH5α and sequenced. The recombinant plasmids were digested with *SpeI* and *AscI* (New England Biolabs, Beijing, China), and the products were individually inserted into the pCLCrVA vector to generate pCLCrV-*GhACNAT* and pCLCrV-*GhPDS*.

The four vectors with or without foreign genes were transformed individually into *Agrobacterium tumefaciens* strain GV3101 by electroporation using a Gene Pulser Apparatus (Bio-Rad, Hercules, CA, USA) as described by Huang *et al*., (2009)^55^. Three combinations, pCLCrVA-empty and pCLCrVB (for a negative control), pCLCrV-*PDS* and pCLCrVB (for a positive control), pCLCrV-*GhACNAT* and pCLCrVB (for target gene silencing) were used. Plants were transformed with pCLCrV-*PDS* and the pCLCrVA-empty vectors as controls. The gene sequences and primers for cloning and detection are listed in Table S1 of the supplementary file.

### Plant growth and agroinfiltration

Cotton seedlings were grown in a growth chamber at 28 °C with a 14 h light and 10 h dark cycle. Healthy 2 week old seedlings were infiltrated with different *Agrobacteria* carrying pCLCrVA or one of its derivatives and pCLCrVB. *Agrobacteria* harboring pCLCrVA or one of its derivatives was mixed with an equal volume of *Agrobacteria* harboring pCLCrVB. The mixed *Agrobacteria* solutions were infiltrated into the abaxial side of the cotyledons of the 2-week-old cotton seedlings using syringes without needles. To facilitate infiltration, a few holes were gently punched into the underside of the cotyledons using a syring needle. The *Agrobacteria* preparation and the process of agroinfiltration used has been described by Gu *et al*., (2014)^15^. The growth chambers for the infected plants were kept at 23 °C with a 14/10 h light/dark cycle. The agroinfiltration was repeated at least three times with approximately 30 plants for each vector in the VIGS system.

Plants infiltrated with the pCLCrVA-empty vector and the wild-type C312 were used as controls and did not show any photobleaching during the experiment. Plants infiltrated with the pCLCrV-*PDS* vector showed the typical photobleaching phenotype in newly developed leaves. However, the efficiency of gene silencing was judged by the intensity of photobleaching, which varied in the different organs of the transgenic plants.

### Anther observation and pollen staining

In our experiments, the transgenic *GhACNAT*-silenced plants and the control plants were compared to observe the change in phenotypes. Pollen activity was detected by staining with 13 mM of I_2_-KI. The solution was prepared by adding 12 mM KI to 5 ml ddH_2_O and then dissolving 4 mM of I_2_ in the solution and adding ddH_2_O to 300 ml. The solution was stored in a brown glass bottle. Pollen grains were soaked in the I_2_-KI solution on glass slides for approximately 1 min, and then the shape and color of the pollen grains were observed with a fluorescence stereomicroscope (ZEISS SteREO LuMar.V12, Germany).

### DNA extraction and PCR detection

Total DNA was extracted from the leaves of pCLCrV-inoculated cotton plants using an extraction buffer containing 100 mM Tris-HCl, pH 8.0, 25 mM Na_2_EDTA, 2 M NaCl, 2% (w/v) CTAB, 2% (w/v) PVP and 2% (w/v) β-mercaptoethanol as described^56^. The presence of pCLCrV DNA in infected tissue was detected by PCR using primers specific for either pCLCrV DNA-A (CLCrVA F and CLCrVA R) or pCLCrV DNA-B (CLCrVB F and CLCrVB R). (Primers used in the experiment are listed in Table S3.) PCR was performed at 94 °C for 4 min, followed by 30 cycles of 94 °C for 30 s, 59 °C for 30 s and 72 °C for 1 min. The PCR products were visualized by electrophoresis on 1% (w/v) agarose gels.

### The analysis by cDNA-AFLP of differentially expressed genes influenced by *GhACNAT*-silencing

The transgenic *GhACNAT*-silenced plants with significantly changed phenotypes were confirmed by PCR and expression analysis of the endogenous *GhACNAT* gene and then were selected for further analysis. The stigmas and stamens of C312 plants and transgenic *GhACNAT*-silenced plants were collected for RNA extraction using TRIzol® Plus RNA Purification Kits (Invitrogen, Carlsbad, CA, USA). Double-stranded cDNA was synthesized from 200 ng RNA using an iScriptTM cDNA Synthesis Kit (Quanta BioSciences, Suite 1 Gaithersburg, MD, USA) according to a standard double-stranded cDNA synthesis protocol. The analysis of differentially expressed genes by cDNA-AFLP was performed to screen for the genes affected by *GhACNAT*-silencing. The cDNA-AFLP analysis was carried out as described by Hawkins *et al*., (2005)^57^. The cDNA was digested with the restriction enzymes *Mse*I and *EcoR* I (New England Biolabs, Beijing, China). The digested products were then ligated to adapters with the following sequences. The *Mse*I adapters were 5’-GACGATGAGTCCTGAG-3’ and 3’-TACTCAGGACTCAT-5’. The *EcoR* I adapters were 5’- CTCGTATACTGCGTACC-3’ and 3’-AATTGGTACGCAGTA-5’. Adapter-ligated cDNA was the template for pre-amplification, with PCR parameters of 30 cycles of 30 s at 94 °C, 60 s at 56 °C, and 60 s at 72 °C. The diluted (30-fold) amplified products were used as the templates for selective amplification. The differentially expressed bands between 100 and 200 bp were cloned into the pGEM-T Easy Vector (Promega, Madison, MI, USA), transformed into *E.coli* DH5α and sequenced.

The categorization of the transcript fragments on the basis of their presence or absence (qualitative variation) or differences in the amount (quantitative variation) in the control lines (wild-type C312 and transgenic pCLCrVA-empty plants) and transgenic *GhACNAT*-silenced lines showed that most of the variation was quantitative. The transcript fragments represented genes controlling different biological processes. The expression levels of *GhACNAT* and differentially expressed genes in the same biological pathway were measured in the control plants and the transgenic *GhACNAT*-silenced plants.

### RNA extraction and quantitative real-time PCR (qRT-PCR) analysis

Total RNA was extracted from fresh leaves or other tissues and organs (roots, stems, bracts, petals, stamens and stigmas, and fibers of different development stages) using a TRIzol® Plus RNA Purification Kit according to the manufacturer’s instructions (Invitrogen, Carlsbad, CA, USA) and treated extensively with RNase-free DNase I. Double-stranded cDNA was synthesized from 100 ng RNA using an iScriptTM cDNA Synthesis Kit (Quanta BioSciences, Suite 1 Gaithersburg, MD, USA) according to a standard double-stranded cDNA synthesis protocol and then diluted 100-fold for qRT-PCR. The qRT-PCR included 5 μl cDNA template (diluted 1/100), 5 μl primers (2.4 μM), and 10 μl SYBR green mixture (GoTaq qPCR Master Mix, Promega, Madison, WI, USA) and was performed in an Eppendorf real-time PCR instrument (Mastercycler ep realplex, Hamburg, Germany). The incubation conditions were 95 °C for 1 min, followed by 40 cycles at 95 °C for 15 s, 60 °C for 30 s, 95 °C for 15 s and 60 °C for 15 s. The specificity of the amplified PCR product was determined by melting curve analysis. The primers for target genes were designed using Premier5 software (Premier Biosoft, Palo Alto, CA, USA) and are listed in Table S3 of the supplementary file.

The genes for the male sterility involved in JA biosynthesis, including *AtOPR3*, *AtDAD1*, *AtMS35*, *AtGPAT1, AtAOS*, and *AtAIF*^22^,^23^,^24^,^25^,^26^ as well as genes for JA biosynthesis catalyzed by several enzymes, including lipoxygenase (LOX), allene oxide synthase (AOS), allene oxide cyclase (AOC), and 12-oxo-phytodienoic acid reductase (OPR) in *A. thaliana*^19^,^20^,^21^ were used as references for the selection of homologous genes in cotton (Table S4). We obtained homologous genes in cotton by BLAST in NCBI (http://www.ncbi.nlm.nih.gov/) and CGP (Cotton Genome Project, Institute of Cotton Research of CAAS, http://cgp.genomics.org.cn/page/species/index.jsp). The expression levels of the homologous genes in cotton were analyzed in transgenic *GhACNAT*-silenced plants and in C312 plants.

The cotton *Ubiquitin7* gene (*GhUBQ7*, Gen Bank accession number DQ116441) was used as an internal control for the assays. The expression levels of endogenous genes in cotton were obtained and standardized to the constitutive *GhUBQ7* gene expression level. In each study, three independent experiments were conducted with three independent samples with the same phenotype per experiment.

### JA Measurement

The new leaves and the whole opening flowers of C312 and transgenic *GhACNAT*-silenced plants were collected and immediately frozen in liquid nitrogen, then stored at −80 °C. Based on the methods of JA extraction^58^,^59^,^60^, samples (approximately 150 mg) were ground and extracted with 1 ml pure cold methanol and stirred overnight at 4 °C in the dark. The suspension was centrifuged at 13.8 g for 15 min at 4 °C, and 900 μl of the supernatant was carefully transferred to a new 1.5-ml tube. The pellet was mixed with 1 ml pure cold methanol, shaken for 4 h at 4 °C, and centrifuged. The two supernatants were combined and dried by a flow of nitrogen gas at room temperature. The residue was dissolved in 200 μl pure methanol. Before GC-MS, derivatization was necessary for JA. The formation of MeJA using trimethylsilyldiazomethane was performed according to the methods in Schmelz *et al*., (2004)^58^. The GC-MS settings have been described^58^,^61^,^62^,^63^ and were used with little modification.

An Agilent 7890 A gas chromatograph (Santa Clara, CA, USA) equipped with a split/splitless injector (splitless mode, 250 °C, injection volume 1 μl) was interfaced to an Agilent 5973C mass spectrometer (Santa Clara, CA, USA) operated in chemical ionization (CI) mode with isobutene as the ionization gas. Compounds were separated on an HP-5 MS (30 m × 0.25 mm × 0.25 μm) column held at 50 °C for 1 min after injection, and then the temperature was programmed at 15 °C (1 min) to 250 °C (10 min), with helium as the carrier gas (1.2 ml /min) (Fig S3).

### The transgenic *GhACNAT*-silenced plant treated by exogenous MeJA

The anther indehiscence phenotype of the *GhACNAT*-silenced plants was rescued by exogenous MeJA. The buds of 3 to 5 days-before-opening in transgenic *GhACNAT*-silenced plants were sprayed with methyl jasmonate (MeJA solution). Different gradient concentrations with 100 μM, 500 μM, 1000 μM dissolved in 0.05% (v/v) Tween-20 were sprayed on buds and leaves according to the description^22^,^23^,^24^. On the opening day of the flower, the productive organs were investigated and analyzed.

## Additional Information

**How to cite this article**: Fu, W. *et al*. Acyl-CoA N-acyltransferase influences fertility by regulating lipid metabolism and jasmonic acid biogenesis in cotton. *Sci. Rep*. **5**, 11790; doi: 10.1038/srep11790 (2015).

## Supplementary Material

Supplementary Information
