# Supplementary material for: Acyl-CoA N-acyltransferase influences fertility by regulating lipid metabolism and jasmonic acid biogenesis in cotton
Source: Sci Rep. 2015 Jul 2;5:11790. doi: 10.1038/srep11790 (PMC4488762; doi:10.1038/srep11790)
Supplement: Supplementary Information [file srep11790-s1.doc]

**Acyl-CoA N-acyltransferase influences fertility by regulating lipid metabolism and jasmonic acid biogenesis in cotton**

Wenfeng Fu^1^, Ying Shen^1^, Juan Hao^1^, Jianyong Wu^2^, Liping Ke^1^, Caiyun Wu^1^, Kai Huang^1^, Binglun Luo^1^, Mingfeng Xu^1^, Xiaofei Cheng^1^, Xueping Zhou^3^, Jie Sun^4^, Chaozhu Xing^2*^, Yuqiang Sun^1*^

1 College of Life and Environmental Science, Hangzhou Normal University, Hangzhou, 310016, Zhejiang, China

2 State Key Laboratory of Cotton Biology, the Institute of Cotton Research of CAAS, Anyang, 455000, Henan, China

3 State Key Laboratory for Biology of Plant Diseases and Insect Pests, Institute of Plant Protection of CAAS, Beijing, 100193, China

4 The Key Laboratory of Oasis Eco-agriculture, College of Agriculture, Shihezi University, Shihezi, 832000, Xinjiang Province, China

* Correspondence and requests for materials should be addressed to Y.S. (email: sunyuq1109@hotmail.com) and C.X. (email: xingcz@cricaas.com.cn).

Supplementary files

**g**

**f**

**e**

**d**

**c**

**b**

**h**

**a**


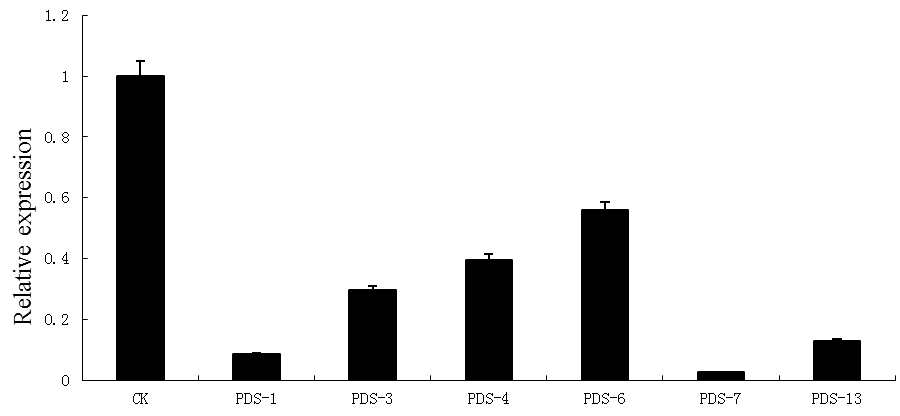
Figure S1 The morphological changes and *GhPDS* gene expression analysis in transgenic *GhPDS*-silencing plants and *G.hirsutum* cv. C312.

**i**

**

**

**

**

**

**

(a,b) the photo-bleaching phenotype firstly appeared in pCLCrV-*PDS*-infected cotton plants after 3-weeks of infection. (c) the phenotype of cotton leaves (pCLCrV-*PDS*, pCLCrV-*GhACNAT*, C312, pCLCrV-empty) in different development stages in the anthersis. (d,e) the different phenotypes of the stems in C312 and *GhPDS*-silencing plants. (f,g) the photo-bleaching in the bracts and the new leaves of *GhPDS*-silencing plants. (h) The obvious different phenotype of photo-bleaching between the up and down leaves of *GhPDS*-silencing plants. (i) Relative expression level of *GhPDS* in leaves from *GhPDS*-silence and C312 plants by qRT-PCR. *Ubiquitin7* gene (*GhUBQ7*) was used as an internal control. The values are the means for three replicates (samples of each line collected on the 20, 35 and 50 day of post-infection). The asterisks indicate statistically significant differences between the transgenic *GhACNAT*-silence and WT C312 plants (*P<0.05, **P<0.01, Student’s t-test).

Table S1 The phenotypes changed in reproductive organ in transgenic *GhACNAT*-silence plants and C312 plants.

| Plant | Length of filaments (mm) | Number of anthers |
| --- | --- | --- |
| CK (C312) | 5.83±0.72^a^ | 35.3±8.6^a^ |
| Line 36-8 | 1.65±0.21^d^ | 22.25±1.7^c^ |
| Line 36-12 | 2.65±0.78^bc^ | 29.25±3.7^b^ |
| Line 36-16 | 3.35±0.25^b^ | 18.75±1.5^d^ |

Means followed by the same letters within a column do not differ significantly at P< 0.05 according to Duncan’s multiple range test.

(n=15 for CK, n=10 for each GhACNAT-silence line to count length of filaments; n=7 for CK, n=4 for each GhACNAT-silence line to count number of anthers)

Table S2 The phenotypes changed in reproductive organ in transgenic *GhACNAT*-silence plants and C312 plants treated by exogenous MeJA.

| Conc. of exogenous MeJA | Plant | Number of plants treated | Number of buds treated | Total number of anther indehiscence |
| --- | --- | --- | --- | --- |
| 100uM | CK (C312) | 3 | 8 | 1 |
|  | *GhACNAT*-silenced | 4 | 10 | 16 |
| 500uM | CK (C312) | 4 | 10 | 0 |
|  | *GhACNAT*-silenced | 3 | 10 | 0 |
| 1000uM | CK (C312) | 5 | 12 | 0 |
|  | *GhACNAT*-silenced | 5 | 14 | 1 |

Table S3 Primers used in the experiments.

| Usage | Primer name | Sequence (5’-3’) |
| --- | --- | --- |
| *GhACNAT* full length cDNA cloning | H36 F | CTATGGCGGCAGCACTCA |
|  | H36 R | TGCACGATGGCTTTTCAA |
| Fragment of *GhACNAT* for pCLCrVA vector | V36 F | **GGACTAGT**CCGACAAAGGGCAAGAAGGT |
|  | V36 R | **TTGGCGCGCC**GCAATGATATGGCTCGGCAT |
| Fragment of *GhPDS* for pCLCrVA vector | VPDS F | **GGACTAGT**GCCTGAAGACTGGAGAGAGATTT |
|  | VPDS R | **TTGGCGCGCC**GCTTTACTCTGATCCGCAGATATT |
| qRT-PCR for *GhUBQ7* expression | GhUBQ7 F | GAAGGCATTCCACCTGACCAAC |
|  | GhUBQ7 R | CTTGACCTTCTTCTTCTTGTGCTTG |
| qRT-PCR for *GhACNAT* expression | T36F | CGAAGCGGCTATCGAGTTGA |
|  | T36R | AATTCCCCTATGGCCTGCTT |
| qRT-PCR for *GhPDS* expression | TPDSF | CGTGGCTCTATGGGTCTTGC |
|  | TPDSR | CTGGTCTGAGGGTGGTCTGC |
| Detection of pCLCrVA vector | A-F | ATTTTGCGCCTGACTAGCCT |
|  | A-R | CGAATTTTCAACGTTGCATACA |
| Detection of pCLCrVB vector | B-F | ATGTACAGTTTAAAGAGTAGACG |
|  | B-R | ATTATCCAATATAATCAAGGTCATAC |
| qRT-PCR for gene expression in JA biosynthesis pathway | GhAIF-F | CAAATGAAGGAACTGAACAAGACC |
|  | GhAIF-R | TGGAGGGAACTCTGAGGCAAA |
|  | GhSERK-F | GTATGACACCGACTGAGCGATTG |
|  | GhSERK-R | GATCATGCAAATAAGAAAGACCCC |
|  | GhDAD1-F | GCTCTGGCTACTCTGACCGCTT |
|  | GhDAD1-R | ACTATTCTCAACACTTTCGTCCCTT |
|  | GhLOX1-F | TGATGGACTGGAGATTTGGTTTG |
|  | GhLOX1-R | GCTTGAAGTTCAGGGTCTTGTTG |
|  | GhACO1-F | CTCCCAAGCCCACAAAAGTT |
|  | GhACO1-R | ACAGAGTCCAGCCGTGATGC |
|  | GhOPR3-F | TGGAAGATGATTGTGGATGCTG |
|  | GhOPR3-R | GAGATGGGTTTGTTTGTTGAGGA |
| qRT-PCR for gene expression in lipid biosynthesis and metabolism pathway | GhWRI1-F | CAGCAAGAACGGCAGGAAAA |
|  | GhWRI1-R | TCCGGAATCCATGCAGAAACT |
|  | GhPKp2-F | TCGTCCTTCTGGCACTATTTTTG |
|  | GhPKp2-R | CCACCTCTTCTCCTTCCTTCACC |
|  | GhPDH-Ea-F | AGAGCTAGGAGAGGAGAAGGTCCA |
|  | GhPDH-Ea-R | GAGGTATTTTTTCAGTGCTGTGATG |
|  | GhMAT-F | GACCCAGCAGTTTCAAGATTGG |
|  | GhMAT-R | AAGTTTTCACCGTGGTTTCCC |
|  | GhKASI-F | TTTTTGGGAACGATGTGGATG |
|  | GhKASI-R | AACCGAGTAGGAAACTTGGAAGC |
|  | GhKASIII-F | CACGGTTAGACTTCCCCCAGG |
|  | GhKASIII-R | GCTTCACCTTTCCGCTTCG |
|  | GhENR-F | TATGGTGGAGGCATGAGTTCAG |
|  | GhENR-R | CTTTCTGTAGGGGTGCGTTAGC |
|  | GhGPDH-F | GTTGGCTAAAGGGGGACTGAG |
|  | GhGPDH-R | TGGTTTGTTTTCTTCGGGGTG |
|  | GhFATA-F | ATCACGTTAGATTATAGGCGGGA |
|  | GhFATA-R | CAACAGATGCAAAAACTTGGGG |
| qRT-PCR for gene expression in glycerolipid biosynthesis and metabolism pathway | GhGPAT2-F | CTTCATCATGAACCCACGTCC |
|  | GhGPAT2-R | GCCTTCCCAATCTCACTCTGC |
|  | GhGPAT3-F | AACACGAGACCGAAAAACAGACG |
|  | GhGPAT3-R | CTCCGCAAACAAGGGACTAAAT |
|  | GhGPAT4-F | TGAATCCAAGGCCAACATACG |
|  | GhGPAT4-R | AACCCCAGGACATCACCCAAC |
|  | GhGPAT8-F | AGGCTCCTATCTCCAATCCCA |
|  | GhGPAT8-R | CGAACAAAGCACTGAACCTCAAC |
|  | GhLPAAT-F | ACTGCCATATTTCTTTTTCCCATT |
|  | GhLPAAT-R | AAATCACCTAACTTCCCATCCTTACT |
|  | GhFAD3-F | ACGCGATTACTGCGATTCACC |
|  | GhFAD3-R | AAGTTTCCTCTCTTGGGGGCT |

Note: the adapter containing restriction enzyme cutting site shown in bold letters.

Table S4 The genes in *A. thaliana* and homologous genes in *G. raimondii* related to lipid, phospholipid and JA biosynthesis measured in the experiments.

| Gene name | locus | Gene name | locus |
| --- | --- | --- | --- |
| *GhAIF* | [Cotton_D_gene_10027600](http://cgp.genomics.org.cn/page/species/mapview.jsp?dbKey=cotton_d&refId=4723&start=33294580&end=33298447) | *AtAIF* | AT3G10500 |
| *GhDAD1* | [Cotton_D_gene_10014954](http://cgp.genomics.org.cn/page/species/mapview.jsp?dbKey=cotton_d&refId=4718&start=38615299&end=38616588) | *AtDAD1* | AT2G44810 |
| *GhLOX1* | [Cotton_D_gene_10018237](http://cgp.genomics.org.cn/page/species/mapview.jsp?dbKey=cotton_d&refId=6147&start=938022&end=941625) | *AtLOX1* | AT1G55020 |
| *GhAOC1* | [Cotton_D_gene_10008831](http://cgp.genomics.org.cn/page/species/mapview.jsp?dbKey=cotton_d&refId=4718&start=19605831&end=19606672) | *AtAOC1* | AT3G25760 |
| *GhOPR3* | [Cotton_D_gene_10037325](http://cgp.genomics.org.cn/page/species/mapview.jsp?dbKey=cotton_d&refId=4723&start=3981095&end=3983881) | *AtOPR3* | AT2G06050 |
|  |  |  |  |
| *GhWRI1* | [Cotton_D_gene_10029828](http://cgp.genomics.org.cn/page/species/mapview.jsp?dbKey=cotton_d&refId=4727&start=41032536&end=41036870) | *AtWRI1* | AT3G54320 |
| *GhPKp2* | [Cotton_D_gene_10017468](http://cgp.genomics.org.cn/page/species/mapview.jsp?dbKey=cotton_d&refId=9034&start=1486225&end=1493073) | *AtPKp2* | AT5g52920 |
| *GhPDH-E1α* | [Cotton_D_gene_10015943](http://cgp.genomics.org.cn/page/species/mapview.jsp?dbKey=cotton_d&refId=4718&start=34258245&end=34259874) | *AtPDH-E1α* | AT1g01090 |
| *GhMAT* | [Cotton_D_gene_10012184](http://cgp.genomics.org.cn/page/species/mapview.jsp?dbKey=cotton_d&refId=4722&start=3171673&end=3174645) | *AtMAT* | AT2g30200 |
| *GhKASI* | [Cotton_D_gene_10040721](http://cgp.genomics.org.cn/page/species/mapview.jsp?dbKey=cotton_d&refId=4717&start=32705322&end=32710101) | *AtKASI* | AT5g46290 |
| *GhKASIII* | [Cotton_D_gene_10015449](http://cgp.genomics.org.cn/page/species/mapview.jsp?dbKey=cotton_d&refId=4725&start=43895926&end=43899626) | *GhKASIII* | AT1G62640 |
| *GhENR* | [Cotton_D_gene_10014580](http://cgp.genomics.org.cn/page/species/mapview.jsp?dbKey=cotton_d&refId=6604&start=1195481&end=1199322) | *AtENR* | AT2g05990 |
| *GhGPDH* | [Cotton_D_gene_10004730](http://cgp.genomics.org.cn/page/species/mapview.jsp?dbKey=cotton_d&refId=5302&start=177331&end=180326) | *AtGPDH* | AT2G41540 |
| *GhFATA* | [Cotton_D_gene_10005540](http://cgp.genomics.org.cn/page/species/mapview.jsp?dbKey=cotton_d&refId=6835&start=633891&end=637472) | *AtFATA* | AT3g25110 |
|  |  |  |  |
| *GhGPAT1* | [Cotton_D_gene_10022412](http://cgp.genomics.org.cn/page/species/mapview.jsp?dbKey=cotton_d&refId=4716&start=10046999&end=10048772) | *AtGPAT1* | AT1G06520 |
| *GhGPAT3* | [Cotton_D_gene_10010671](http://cgp.genomics.org.cn/page/species/mapview.jsp?dbKey=cotton_d&refId=5186&start=918401&end=920370) | *AtGPAT3* | AT4G01950 |
| *GhGPAT4* | [Cotton_D_gene_10035603](http://cgp.genomics.org.cn/page/species/mapview.jsp?dbKey=cotton_d&refId=4719&start=42879047&end=42884012) | *AtGPAT4* | AT1G01610 |
| *GhGPAT8* | [Cotton_D_gene_10035603](http://cgp.genomics.org.cn/page/species/mapview.jsp?dbKey=cotton_d&refId=4719&start=42879047&end=42884012) | *AtGPAT8* | AT4g00400 |
| *GhLPAAT* | Cotton_D_gene_10037128 | *AtLPAAT* | AT4G30580 |
| *GhFAD3* | [Cotton_D_gene_10013007](http://cgp.genomics.org.cn/page/species/mapview.jsp?dbKey=cotton_d&refId=4721&start=27157969&end=27159670) | *AtFAD3* | AT2g29980 |

Retention time

a

Time

Retention time

Retention time

b

c

Figure S2 JA measured by gas chromatography-MS linkage analysis in transgenic *GhACNAT*-silenced plants and C312. (a) standard sample of *cis* and *trans* isomers of MeJA at 11.109 of retention time; (b) chromatogram of WT C312 plant; (c) chromatogram of transgenic *GhACNAT*-silenced plant.


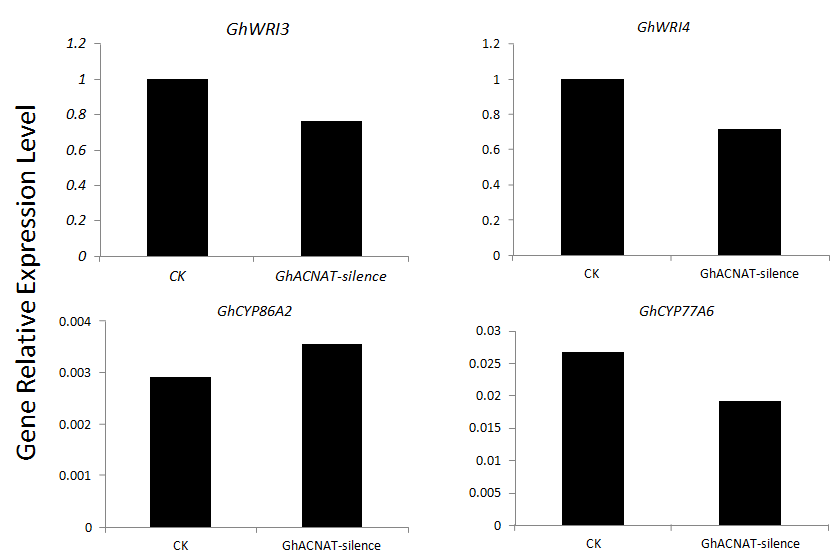


d

c

b

a

Figure S3 gene expression in fatty acid biosynthesis pathway alteration in C312 and transgenic *GhACNAT*-silenced plants stamens.

(a,b) two additional regulators of fatty acid biosynthetic pathway, which are closely related to WRI1 and belong to the APETALA2–ethylene-responsive element binding protein family of transcription factors. (c,d) The relative mRNA levels of another two genes involved in cuticle formation.

Gene sequence information:

1. *GhACNAT* sequence:

CTATGGCGGCAGCACTCAGCTACTGCAAGCCTTCCCCATTCATTGGCCAATTTCCTTCCAACTTTGGGAAGCCAGTATCTCTGCGAAGCATCGAAATCTCGACGCAGGCATCTAGAATCACTGCACTGTTTTGGGGATCTAAGAAGTCTGTGAAGCATCAACCTGTGGATTCTTCTTTGGGGGATTTCACTTTGACAGGGTCAGAAACAGAGGAACTTAAAGAGAACCCGACAAAGGGCAAGAAGGTATCGGTATCGATTATTTCCTCAATTCTGGACGTTTCTTCACATGAATGGGATTCTTGCGCTCTGGATGCTACCGGTCCTGAAAAGTTCAATCCATTTCTTTCTCATGGTTTCCTTTCAAGCTTGGAAGAGACGGGTTGCGCAGTGAAGGAAACAGGATGGATGCCGAGCCATATCATTGCTAAGGATGAATCTGAAAATATTTTAGGTGTTGCTCCTCTCTATCTTAAAAGCCATTCCTATGGTGAATTTGTTTTCGATCATTCTTGGGCAGATGCATATTATAGTTTTGGAGCAAGATATTACCCAAAGTTCCAGTGTTGTGTGCCTTTCACTCCAGTGACTGGTCCTAGGATTTTAGTACGGAATACATCATTCAAGGATCAAGTTTTTGACGTTATAGTCACTGCTCTGAAGGATCTGACAGCGAAGTCTCAGGTTTCCTCTCTGCACATTACTTTCCCATCTGAAGCCGAATGGTACAAACTGAAGGATAGAGGATTCCTACAGAGGATTGGAATGCAATACCACTGGAAGAATCGCAACTATAAAAGTTTTGACGAGTTCTTGATGGACATGAAGCAAAGTAAAAGGAAAAATATCCGTCAAGAGCGCAAAAAGATTCCTGCTCAGGATTTGACAATGAAACGGCTCAGAGGTTATGAAATTAAGGCCAATCACTGGGATTCCTTCTACAAGTTCTACCGGAATACTACTGATAATAAGTGGGGCAGTCCATACCTAACAAGAGATTTCTTTCACGAAATGGGATCAAAGATGGGAGATGATGTGTTACTCGTAGTTGCCGAAAAAAGGGACGAGCTTGTTGCAGGAGCTCTGAATCTCATTGGAGGCGATACTATATATGGACGCTTATGGGGATGTGACCCTCAAGTCTATTATCCGAGCTTGCATTTTGAAGCATGTTATTATCAGGCAATCGAAGCGGCTATCGAGTTGAATCTAAGCACAGTAGAGGCTGGAGCTCAGGGTGAGCATAAGATTCAGCGAGGCTATTTGCCGGTGCCGACTTATAGCTGTCATTACTTTATCGATGAGGGTTTCAAGCAGGCCATAGGGGAATTTCTGGTTCGAGAATCAAATCAGGTTGACCTTGTTATGAAACTATTTCATGAATCTGGTCCCTTTAAGGAGGGCATACACTAACGGAGAGTTGCTGGAAGTTTGATTGAAAAGCCATCGTGCA

The sequence underlined was inserted into pCLCrVA vector for VIGS.

2. *Gossypium hirsutum* phytoene desaturase (PDS) mRNA, partial CDS of 327 base pairs (gi|171188389) for pCLCrVA vector

GCCTGAAGACTGGAGAGAGATTTCATACTTCAAGAAATTAGAGAAATTAGTTGGAGTTCCAGTTATCAACGTTCACATCTGGTTTGATAGGAAATTGAAGAACACCTATGATCATCTACTCTTTAGCAGAAGCCCGCTTTTAAGTGTTTATGCTGACATGTCTGTAACATGTAAGGAATATTACAATCCAAACCAATCCATGTTGGAGTTAGTTTTTGCCCCAGCAGAAGAATGGATTGCATGTAGTGACTCAGAAATTATTGATGCTACAATGAAGGAACTTGCAAAGCTCTTTCCTGATGAAATATCTGCGGATCAGAGTAAAGC
